# Universal capability of 3-ketosteroid Δ^1^-dehydrogenases to catalyze Δ^1^-dehydrogenation of C17-substituted steroids

**DOI:** 10.1186/s12934-021-01611-5

**Published:** 2021-06-23

**Authors:** Patrycja Wójcik, Michał Glanowski, Agnieszka M. Wojtkiewicz, Ali Rohman, Maciej Szaleniec

**Affiliations:** 1 Jerzy Haber Institute of Catalysis and Surface Chemistry Polish Academy of Sciences, Niezapominajek 8, 30239 Krakow, Poland; 2 Department of Chemistry, Faculty of Science and Technology, Universitas Airlangga, Surabaya, 60115 Indonesia; 3 Laboratory of Proteomics, Research Center for Bio-Molecule Engineering (BIOME), Universitas Airlangga, Surabaya, 60115 Indonesia; 4 Laboratory of Biophysical Chemistry, University of Groningen, 9747 AG Groningen, The Netherlands

**Keywords:** 3-ketosteroid dehydrogenase, KSTD, Cholest-4-en-3-one Δ^1^-dehydrogenase, 3-ketosteroids, 1,2-dehydrogenation, Δ^1^-dehydrogenation, Cholest-4-en-3-one, Diosgenone, Cholesterol metabolism

## Abstract

**Background:**

3-Ketosteroid Δ^1^-dehydrogenases (KSTDs) are the enzymes involved in microbial cholesterol degradation and modification of steroids. They catalyze dehydrogenation between C1 and C2 atoms in ring A of the polycyclic structure of 3-ketosteroids. KSTDs substrate spectrum is broad, even though most of them prefer steroids with small substituents at the C17 atom. The investigation of the KSTD’s substrate specificity is hindered by the poor solubility of the hydrophobic steroids in aqueous solutions. In this paper, we used 2-hydroxpropyl-β-cyclodextrin (HBC) as a solubilizing agent in a study of the KSTDs steady-state kinetics and demonstrated that substrate bioavailability has a pivotal impact on enzyme specificity.

**Results:**

Molecular dynamics simulations on KSTD1 from *Rhodococcus erythropolis* indicated no difference in ΔG_bind_ between the native substrate, androst-4-en-3,17-dione (AD; − 8.02 kcal/mol), and more complex steroids such as cholest-4-en-3-one (−﻿ 8.40 kcal/mol) or diosgenone (−﻿ 6.17 kcal/mol). No structural obstacle for binding of the extended substrates was also observed. Following this observation, our kinetic studies conducted in the presence of HBC confirmed KSTD1 activity towards both types of steroids. We have compared the substrate specificity of KSTD1 to the other enzyme known for its activity with cholest-4-en-3-one, KSTD from *Sterolibacterium denitrificans* (AcmB). The addition of solubilizing agent caused AcmB to exhibit a higher affinity to cholest-4-en-3-one (Ping-Pong bi bi K_mA_ = 23.7 μM) than to AD (K_mA_ = 529.2 μM), a supposedly native substrate of the enzyme. Moreover, we have isolated AcmB isoenzyme (AcmB2) and showed that conversion of AD and cholest-4-en-3-one proceeds at a similar rate. We demonstrated also that the apparent specificity constant of AcmB for cholest-4-en-3-one (k_cat_/K_mA_ = 9.25∙10^6^ M^−1^ s^−1^) is almost 20 times higher than measured for KSTD1 (k_cat_/K_mA_ = 4.71∙10^5^ M^−1^ s^−1^).

**Conclusions:**

We confirmed the existence of AcmB preference for a substrate with an undegraded isooctyl chain. However, we showed that KSTD1 which was reported to be inactive with such substrates can catalyze the reaction if the solubility problem is addressed.

**Supplementary Information:**

The online version contains supplementary material available at 10.1186/s12934-021-01611-5.

## Background

**Fig. 1:**
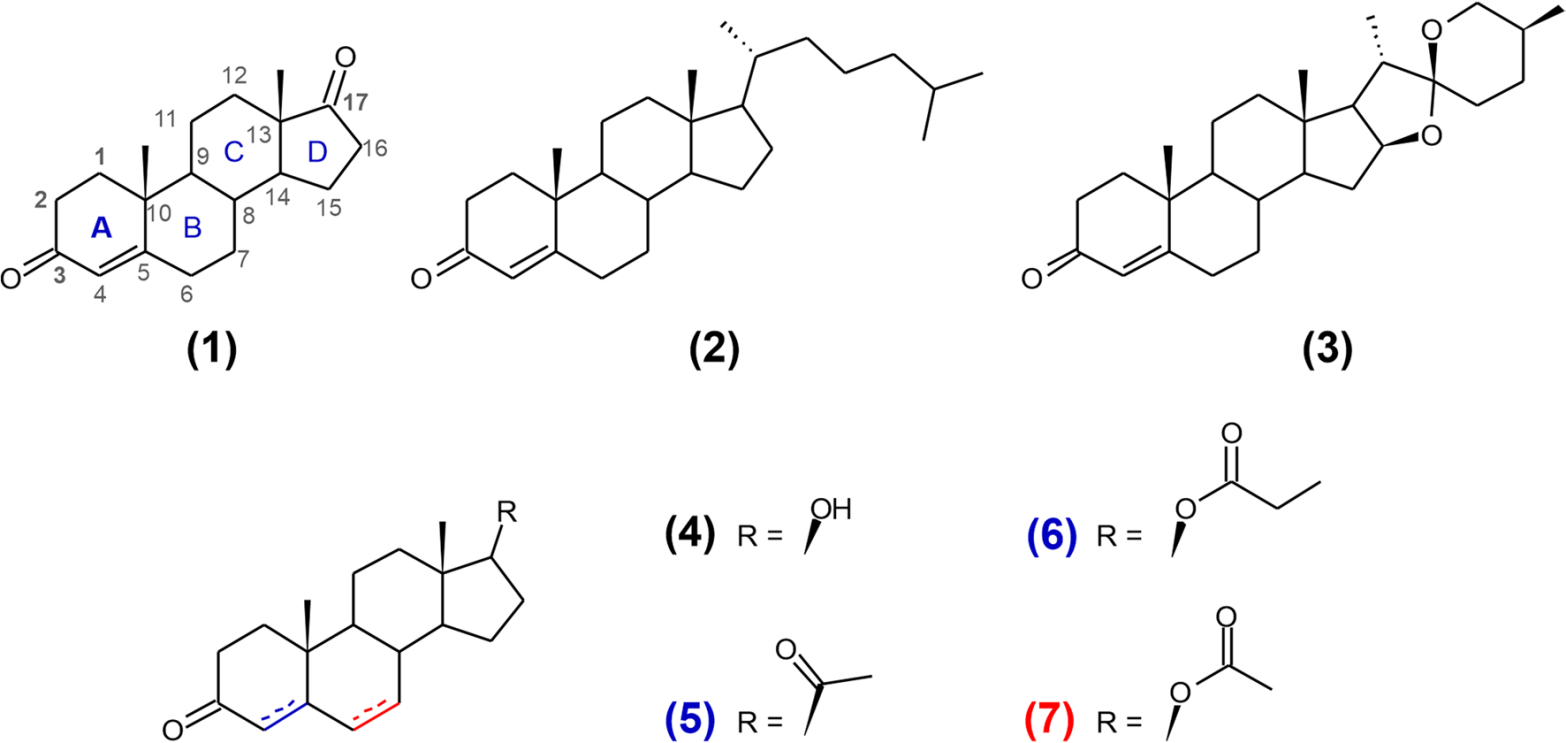
Structures of compounds tested in this study as substrates: (**1**) androst-4-en-3,17-dione, (**2**) cholest-4-en-3-one, (**3**) diosgenone, (**4**) androstanolone, (**5**) progesterone, (**6**) testosterone propionate and (**7**) 6-dehydrotestosterone acetate

Steroids are widespread compounds in the natural environment. They are essential components of eukaryotic i.e., animal, yeast and fungi and plant, cells. Cholesterol, the parent substance of all sterols, is one of the most commonly occurring. Hence bacteria have evolved to use it as a carbon and energy source [1]. The microbial degradation of cholesterol proceeds under either aerobic or anaerobic conditions and the current stage of knowledge was recently reviewed by Rohman et al*.* [2]. 3-Ketosteroid Δ^1^-dehydrogenases (KSTDs) initiate the crucial step of the degradation – steroid nucleus decomposition and as a result are found in aerobic, facultative anaerobic and strictly anaerobic microorganisms [3–5]. KSTDs catalyze regio- and stereoselective dehydrogenation between C1 and C2 atoms of steroid ring A (Fig. 1) [6]. The stage at which the double bond is introduced during cholesterol degradation is still debated and may differ between bacterial species [2].

The wide KSTDs substrate spectrum, the regioselectivity of the catalyzed reaction and mild conditions of the synthesis make those enzymes widely applied for the biologically active Δ^1^-dehydrosteroids production [2]. The 1,2-dehydrogenation process can be carried out with either a whole-cell system (native and recombinant bacteria are used) or isolated enzymes [7, 8]. The poor solubility of steroids in aqueous solutions is usually circumvented by the application of pseudo-crystallofermentation technique (i.e., steroid substrate in the solid-state) or by the addition of organic solvents and various solubilizing agents, e.g., vegetable oils, phospholipids, glycols, dextrans or cyclodextrins [8–15]. The biocompatibility of cyclodextrins makes them valuable solubilizing agents for scale-up microbial steroid biotransformation. Importantly, they do not exert a detrimental influence on the stability of the purified enzymes [16, 17].

Although KSTDs substrate scope is generally broad, most of the enzymes prefer steroids with small substituents on the C17 position [2]. Only a few of them exhibited activity towards more branched substrates, such as cholest-4-en-3-one, 3-oxo-5β-cholan-24-oic acid, the methyl ester of 3-oxo-4-cholen-24-oic acid, cortisone acetate, hydrocortisone acetate or 21-acetoxy-pregna-4,9(11),16-triene-3,20-dinone [18–23]. The comparative study of substrate specificity is additionally hindered by poor or very bad solubility of steroids in the water phase in which all kinetic enzyme assays are conducted [8].

3-Ketosteroid Δ^1^-dehydrogenase from *Streolibacterium denitrificans* (AcmB) is a good example of the enzyme for which the native substrate remains an open question [24]. So far two 3-ketosteroids have been proposed as the AcmB natural substrates. Cholest-4-en-3-one (**2** in Fig. 1) was initially proposed as the AcmB native substrate [21, 25, 26]. However, subsequent research demonstrated a high affinity of the enzyme for C19 – C21 steroids and indicated that AcmB is located on the cytoplasmic side of the inner membranes of *S. denitrificans* [8, 27]*.* These results suggested that androst-4-en-3,17-dione (AD; **1**) may be the enzyme native substrate [28]. Nevertheless, AcmB’s ability to catalyze Δ^1^-dehydrogenation of steroids with the aliphatic side chain on the C17 position seemed interesting and quite unusual.

In this work, we demonstrate that substrate bioavailability can significantly influence the kinetic results and lead to misinterpretation of enzyme specificity. We used 2-hydroxpropyl-β-cyclodextrin (HBC) as a solubilizing agent to study the steady-state kinetics for AcmB and 3-ketosteroid Δ^1^-dehydrogenase from *Rhodococcus erythropolis* SQ1 (KSTD1) with AD and cholest-4-en-3-one. In contrast to previous reports [2, 29], we have demonstrated never reported before KSTD1 activity towards cholest-4-en-3-one and even more complex 3-ketosaponin – diosgenone (**3**). Moreover, we proved that AcmB indeed exhibits a significantly higher affinity to cholest-4-en-3-one than to AD while KSTD1 indeed prefers smaller ketosteroids.

## Methods

### Materials

All chemicals were purchased from Sigma-Aldrich, Tokyo Chemical Industry, Carl Roth or BioShop unless otherwise specified. (25*R*)-Spirost-1,4-dien-3-one (diosgenone) was a gift of Jacek Morzycki from the University of Bialystok.

### Proteins expression and purification

The AcmB was cloned, expressed and purified as described previously at Wojtkiewicz et al*.* work [8].

A gene acmB2 was amplified from genomic *S. denitrificans* DNA using 0.04 µM of primers: forward (5’-TACTTCCAATCCAATGCTATGAGCGGAGAAACTTTCG-3’) and reverse (5’-TTATCCACTTCCAATTCATACCGCGCTCCG-3’), 1 U Platinum™ Taq DNA Polymerase High Fidelity (ThermoFisher), 2.4% dimethyl sulfoxide (DMSO), 200 µM dNTP mix, High Fidelity PCR Buffer, 2 mM MgSO_4_, 160 ng of template DNA and thermocycler (BioRad) program as follows: 94 °C for 2 min, 32 cycles of (94 °C for 15 s, 63 °C for 30 s, 68 °C for 120 s) with a final elongation at 68 °C for 10 min. After gel electrophoresis a band of size ~ 1.7 kbp was excised from agarose gel and purified using Gel-Out kit (A&A Biotechnology) according to the manufacturer’s instructions. Next, gene encoding AcmB2 (GenBank: SMB21450.1) was cloned into a pMCSG7 vector. 25 µL pMCSG7 vector (200 ng) was treated with 2.5 µL SspI in a total of 50 µL 1 × Tango Buffer (Thermo Scientific) supplemented with 4 mM dithiothreitol (DTT) for 1.5 h at 37 °C. The linearized, gel-purified vector was prepared for ligase-independent cloning. Namely, 200 ng of pMCSG7 vector were incubated in 40 µL 1 × T4 Polymerase buffer (Novagen) supplemented with 4 mM DTT, 1 µL of T4 Polymerase (Novagen) and 200 µM dGTP (Promega) for 40 min at room temperature, which was followed by a 10 min inactivation of the polymerase at 75 °C. Similarly, the acmB2 gene was prepared, however, the nucleotides were substituted with dCTP (Promega). Then 3.5 µL of vector preparation was reacted with 6 µL of gene preparation and incubated on ice for 3 h. The mixture was transformed into *Escherichia coli* XL10-Gold Ultracompetent Cells (Agilent Technologies) and the cells were grown overnight in 2% (w/v) Lennox Broth (LB) supplemented with ampicillin 100 μg mL^−1^ and 34 μg mL^−1^ chloramphenicol. Afterward, the plasmid was extracted using Plasmid Miniprep DNA Purification Kit (EURx) according to the manufacturer’s instructions, sequenced by the Sanger sequencing method (Genomed, Poland) and transformed into calcium chloride chemically competent *E. coli* BL21(DE3)Magic (Creative Biolabs). Bacteria cultivation, harvesting and disruption, as well as enzyme purification were conducted as described for AcmB [8].

A gene encoding 3-ketosteroid Δ^1^-dehydrogenase from *R.erythropolis* (KSTD1) was cloned into pET15b vector [30] and used to transform *E. coli* BL21(DE3)Magic cells. An overnight culture of the transformed cells was grown in 2% (w/v) LB supplemented with 100 μg mL^−1^ ampicillin and 50 μg mL^−1^ kanamycin at 37 °C, 180 rpm. The preculture was hundred times diluted in 1 L of fresh LB containing 0.5 M D-sorbitol and antibiotics and grown at 37 °C, 180 rpm. When the OD_600_ reached 0.6, the temperature was reduced to 16 °C and the culture was induced with 100 μM isopropyl β-D-1-thiogalactopyranoside (IPTG). After 48 h the *E. coli* cells were centrifuged for 1 h at 4500 g, 4 °C. The harvested cells were resuspended in a 1:5 ratio (w/v) in the 50 mM Tris–HCl pH 8.5 buffer, 100 mM NaCl, 10% (w/v) glycerol, 5 mM β-mercaptoethanol (BME) and 10 mM imidazole and supplemented with 100 μM phenylmethylsulfonyl fluoride (PMSF). The cells were disrupted by sonication (Sonics Vibra-Cell VCX500, 3 s on, 5 s off, 5 min, 40% amplitude, 150 000 J). The cell-free extract was obtained by centrifugation at 40 000 g, for 1 h at 4 °C. Purification of KSTD1 was carried out on 5 mL HisTrap HP (GE Healthcare) column using FPLC system (BioRad NGC Quest 10 Plus) and linear imidazole gradient 10 – 300 mM in the previously described buffer. The fractions that absorbed UV-light at wavelength 450 nm were collected, desalted using dialysis tubing with 10 kDa cut-off (Thermo Fisher Scientific) according to the manufacturer’s instructions and stored at –20 °C.

The concentration of purified AcmB, AcmB2 and KSTD1 was determined using a free FAD extinction coefficient of 11 300 M^−1^ cm^−1^ at 450 nm.

### Spectrophotometric activity assay

The steady-state kinetic parameters (K_m_, V_max_, k_cat,_ k_cat_/K_m_) for steroids were determined in a spectrophotometric activity assay using UV-2700 spectrophotometer (Shimadzu) in 0.5 mL quartz cuvettes with 10 mm path length. The measurements were carried out in 0.1 M K_2_HPO_4_/KH_2_PO_4_ buffer pH 6.5 (AcmB) or 0.1 M K_2_HPO_4_/KH_2_PO_4_ buffer pH 8.0 (KSTD1) with 0.029 – 0.174 mM 2,6-dichloroindophenol (DCPIP), 2% (w/v) 2-hydroxypropyl-β-cyclodextrin (HBC; 14.54 mM), 5 to 500 μM concentrations of the steroids dissolved in 2-methoxyethanol (EGME, the final concentration 2% (v/v)) and 0.30 μM of AcmB or 0.23 μM of KSTD1, respectively. The reduction of DCPIP was followed at 700 nm (ε_pH 6.5, HBC_ = 10 691 M^−1^ cm^−1^ or ε_pH 8.0, HBC_ = 11 627 M^−1^ cm^−1^). All measurements were performed in triplicates at 30 °C. The linear slopes were obtained with linear regression fitted to the initial parts (5 s) of the kinetic curves. The Ping-Pong bi bi (non-sequential) mechanism model was fitted to the obtained data for AcmB. The linear function was fitted to the data obtained for KSTD1. All data processing was conducted using OriginPro 2019 software.

### HPLC analysis

To confirm KSTD1 activity with diosgenone mini reactor tests were performed. The reaction mixture (1 mL) consisted of 50 mM Tris–HCl buffer pH 8.0, 0.15 mM DCPIP, 2% (w/v) HBC, 0.1 mM diosgenone in dioxane (the final concentration 2% (v/v)) and 87.7 nM KSTD1. The reactions were carried out in duplicate at 30 °C, in the thermoblock at 800 rpm for 20 min. To determine the conversion of the substrate, the reaction mixtures were diluted 1:1 with acetonitrile (ACN), centrifuged at 15 000 g for 5 min and analyzed with HPLC–DAD (Agilent 1100) according to [8].

To confirm AcmB2 activity with AD, cholest-4-en-3-one and diosgenone mini reactor tests were performed. The reaction mixture (1 mL) consisted of 50 mM Tris–HCl buffer pH 8.0, 0.15 mM DCPIP, 2% (w/v) HBC, 0.1 mM of substrate in EGME (the final concentration 2% (v/v)) and 6.55 nM AcmB2 for AD and cholest-4-en-3-one or 65.5 nM for diosgenone. The reaction was carried out in duplicate at 30 °C, in the thermoblock at 800 rpm for 960 h. To determine the conversion of the substrates, the samples were prepared and analyzed as described above.

### Determination of HBC/steroid stability constant

The stability constants (K_S_) of the inclusion complex between HBC and steroids were determined according to the modified protocol described by Ma et al.[31]. The mixtures of various concentrations of HBC (from 0 to 98 mM), 2% (v/v) EGME and the double molar excess of AD or cholest-4-en-3-one were incubated in the thermoblock at 30 °C, 1000 rpm for 48 h. Afterward, the suspensions were filtered through 0.45 μm membrane filters, diluted 0–200 times with ACN, centrifuged at 15 000 g for 15 min, and concentration of soluble steroid was quantified by HPLC–DAD using calibration on external standards [8]. The linear function was fitted to the data obtained for AD. The stability constant for AD:HBC 1:1 complexes (K_1:1_) was calculated from Eq. (1), where S_0_ is an *y*-intercept [32, 33].

1 $${\mathrm{K}}_{1:1}=\frac{\mathrm{s}\mathrm{l}\mathrm{o}\mathrm{p}\mathrm{e}}{{\mathrm{S}}_{0}(1-\mathrm{s}\mathrm{l}\mathrm{o}\mathrm{p}\mathrm{e})}$$

The quadratic function ($[\mathrm{S}]=\mathrm{a}{[\mathrm{H}\mathrm{B}\mathrm{C}]}^{2}+\mathrm{b}\left[\mathrm{H}\mathrm{B}\mathrm{C}\right]+{\mathrm{S}}_{0}$) was fitted to the data attained for cholest-4-en-3-one. The stability constants for cholest-4-en-3-one, HBC 1:1 (K_1:1_) and 1:2 (K_1:2_) complexes, were calculated from the Eq. (2) and (3), respectively [33].

2 $${\mathrm{K}}_{1:1}=\frac{\mathrm{b}}{{\mathrm{S}}_{0}}$$

3 $${\mathrm{K}}_{1:2}=\frac{\mathrm{a}}{{\mathrm{S}}_{0}{\mathrm{K}}_{1:1}}$$

S_0_ refers to the solubility of steroids in 2% (v/v) EGME/water solution (144.2 ± 7.1 μM for AD and 4.8 ± 0.5 μM for cholest-4-en-3-one).

### MD simulations

The structure of KSTD1 was taken from Protein Data Bank (PDB ID: 4c3y [34], resolution 2.3 Å). 1,4-androstadiene-3,17-dione (ANB) and FAD cofactor were already present in the active site of the crystal structure. KSTD1 was determined to be monomeric in solution [34] and, thus, we used the monomeric structure of the enzyme to perform MD simulations. To place structures of androst-4-en-3,17-dione (**1**), cholest-4-en-3-one (**2**) and diosgenone (**3**), androstanolone (**4**), progesterone (**﻿5**), testosterone propionate (**﻿6**) and 6-dehydrotestosterone acetate (**﻿7**) in the enzyme active site, the Kabsch method was used [35] with ANB as a template. The geometry of each substrate was obtained by optimization in the gas phase at B3LYP/6-31G(d,p) level of theory with Gaussian16 [36]. PropKa ver 3.1 [37, 38] and H ++ packages [39] were used to determine the protonation states of titratable amino acids. The optimal pH for enzyme activity was reported to be about 8.0, so this value was selected for model construction. We assumed that Tyr318 is deprotonated due to the mechanistic hypothesis. AMBER parameters for ketosteroids and tyrosyl anion were obtained with Gaussian 16 software package [36] (at B3LYP/6-31G(d,p) level of theory) and ANTECHAMBER program from AmberTools [40]. Parameters for FAD were obtained from the RESP ESP charge DataBase (R.E.DD.B) [41]. A total amount of 33 sodium cations were added to neutralize the system. Finally, the system was soaked in a 93.5 × 76.5 × 72.0 Å^3^ box with TIP3P [42] water molecules.

To prepare the model for MD simulation, the system was optimized with Amber package [43] with ff03 force field [44], heated from 0 to 303 K over 100 ps with NVT conditions (canonical ensemble), and equilibrated for 100 ps with NPT (isothermal-isobaric) ensemble. Finally, 60 ns of NPT molecular dynamic simulation at 303 K was performed.

The ΔG of substrate binding was estimated using the MM-PBSA algorithm in a Poisson Boltzmann (PB) solvation [45] for 500 frames (snapshots from every 10 ps) from selected 5 ns of each MD simulation. Ionic strength was set to 0.15 M. To select a fragment of trajectory, distances describing the position of steroid were calculated: d(O_keto_-OH_Tyr487_), d(H2β-OH_Tyr318_) and d(H1α-N_FAD_). The first of these parameters is describing the hydrogen bond between the 3-keto group of ketosteroid and Tyr487, the next two are related to the postulated mechanism of the catalyzed reaction, which postulates that H2β and H1α are transferred to Tyr318 and FAD respectively. For MMPBSA calculation we selected 5 consecutive ns, during which the sum of the standard deviation of these parameters was minimal.

### Results

#### Theoretical prediction of substrate specificity

**Fig. 2:**
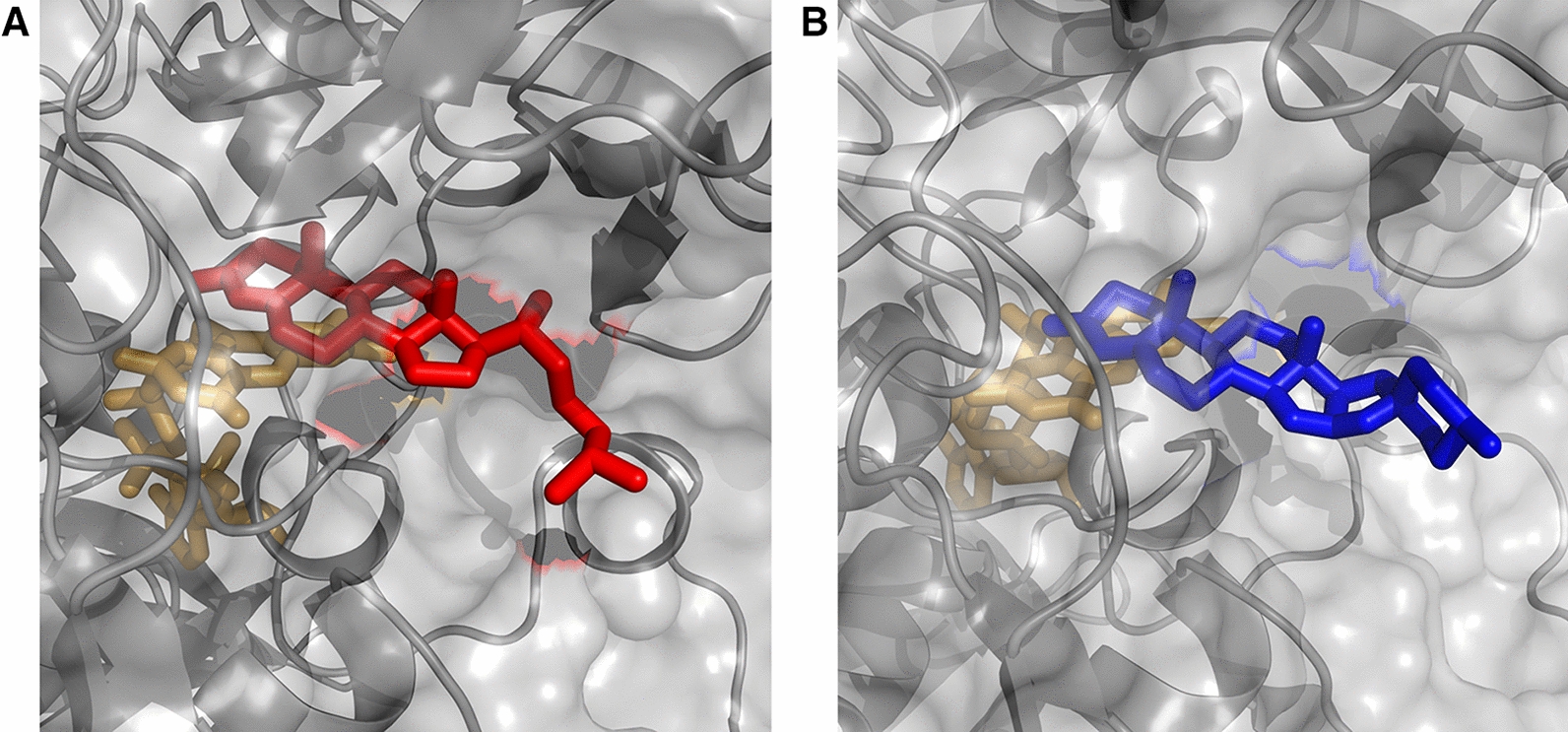
Frame from MD simulation on KSTD1 with **A** cholest-4-en-3-one (red) and **B** diosgenone (blue) in the active site. FAD is marked with light orange (see SI for pdb structures)

In the crystal structure of KSTD1 in complex with the ligand 1,4-androstadiene-3,17-dione (*cf.* **1**), the steroid ring core (gonane ring system) of the ligand was buried in the active site pocket of the enzyme with its D-ring pointing towards the solvent-accessible area [34]. Likewise, the MD simulation of KSTD1:cholest-4-en-3-one complex revealed that the enzyme binds the steroid ring core while the isooctyl substituent at C17 position protrudes out of the active site and generally does not prevent it from the active binding (Fig. 2).

During the simulation, we also did not observe any significant clashes of the isooctyl side chain with the protein which would explain lack of the enzyme activity toward this class of substrate. From the reaction point of view, the most important interactions between substrate and the enzyme were limited to the 3-keto group of steroid ring A and its closest neighborhood (especially vicinity of C1 to FAD and C2 to Tyr318). However, in the literature, cholest-4-en-3-one was reported as inactive in the reaction with KSTD1 [29]. Therefore, we decided to check if the binding free energies calculated for C17-substituted and non-substituted steroids are consistent with the reported experimental results. Free energies of substrate binding were estimated for KSTD1 with MM-PBSA (Table 1). All studied compounds bound favorably to the active site with ΔG_bind_ in the range of − 11.0 to − 7.9 kcal/mol. Interestingly, there was no difference in ΔG_bind_ between the native substrate of KSTD1 from *R. erythropolis,* androst-4-en-3,17-dione (− 8.02 ± 0.13 kcal/mol, **1**), and cholest-4-en-3-one (− 8.40 ± 0.19 kcal/mol, **2).** Based on this result we formed the hypothesis that in principle it should be possible to convert with KSTD1 not only 3-ketosteroids with a degraded side chain (**1, 4, 5–7**) but also steroids such as **2** or even 3-ketosaponins as **3** (Fig. 2).

**Table 1:** The estimated free energy of the binding of steroids to the KSTD1 active site

Ligand	ΔG_bind_ [kcal/mol]	Std. err. of ΔG_bind_ [kcal/mol]
Androst-4-en-3,17-dione (**1**)	− 8.02	0.13
Cholest-4-en-3-one (**2**)	− 8.40	0.19
Diosgenone (**3**)	− 6.17	0.15
Androstanolone (**4**)	− 10.97	0.15
Progesterone (**5**)	− 8.23	0.17
Testosterone propionate (**6**)	− 7.85	0.15
6-Dehydrotestosterone acetate (**7**)	− 8.53	0.15

### HBC/steroid inclusion complex formation

**Fig. 3:**
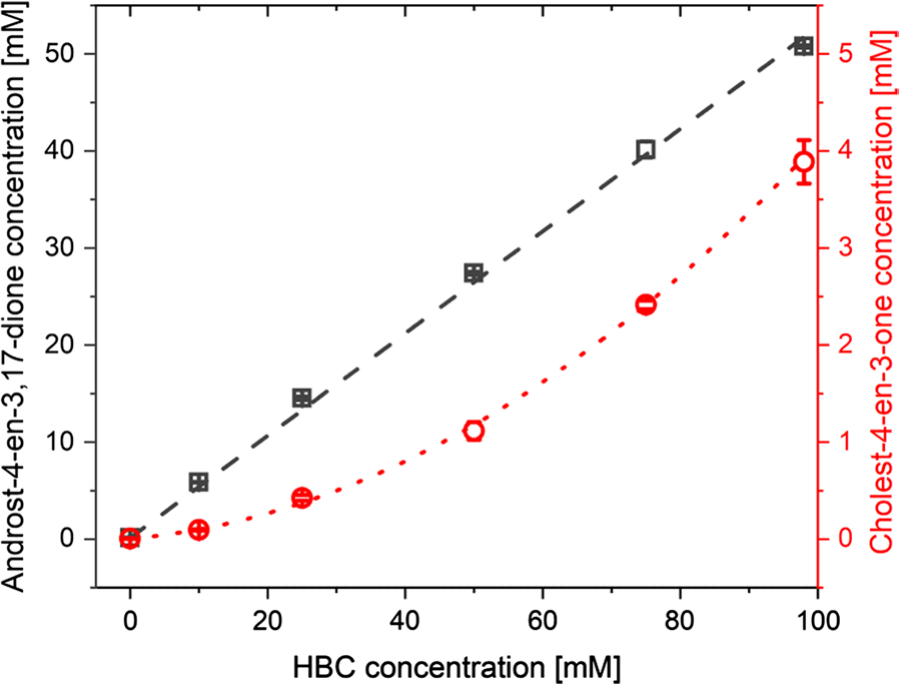
Phase solubility diagrams for androst-4-en-3,17-dione (gray squares) and cholest-4-en-3-one (red circles)

The phase solubility diagrams of AD (**1**) and cholest-4-en-3-one (**2**) with HBC were determined in the HBC concentration range from 0 to 98 mM at 30 °C, i.e., the temperature of catalytic assays (Fig. 3). As expected, the solubility of both steroids increased with higher HBC concentration. However, the solubility of more hydrophobic **2** in 98 mM HBC solution turned out to be over 10-times lower than the AD.

The concentration of AD in the function of HBC concentration was fitted with the linear function (*R*^2^ > 0.998) corresponding to A_L_-type phase diagram. The linear course of phase solubility diagram with the slope less than 1 (0.527 ± 0.009) indicates that HBC with AD forms the 1:1 inclusion complex. Meanwhile, the cholest-4-en-3-one/HBC phase solubility diagram turned out to be the A_P_-type and the data were fitted with quadratic function (R^2^ > 0.998). A_P_-type phase solubility diagram may indicate the formation of higher-order complexes with respect to cyclodextrin at higher HBC concentrations [31, 32]. The fit parameters are presented in Additional file 1: Tables S1–S4, while the values of K_1:1_ and K_1:2_ were shown in Table 2.

**Table 2:** Solubility parameters of steroids in the solution of HBC and 2% EGME at 30 °C

Substrate	Solubility curve type	K_1:1_ [M^−1^]	K_1:2_ [M^−1^]
Androst-4-en-3,17-dione (**1**)	A_L_	7714.4	–
Cholest-4-en-3-one (**2**)	A_P_	1277.1	56.8

The obtained constants were used to calculate the percentage of free and complexed steroids in the reaction medium. For androst-4-en-3,17-dione 99% of the substrate has been complexed by HBC. The ratio of free and complexed (1:1 and 1:2) cholest-4-en-3-one has varied slightly depending on the initial steroid concentration (Additional file 1: Table S2) and averaged 2.9% of the free form, 53.5% of the steroid in 1:1 complex and 43.6% of the steroid in 1:2 complex.

### AcmBs native substrate

**Fig. 4:**
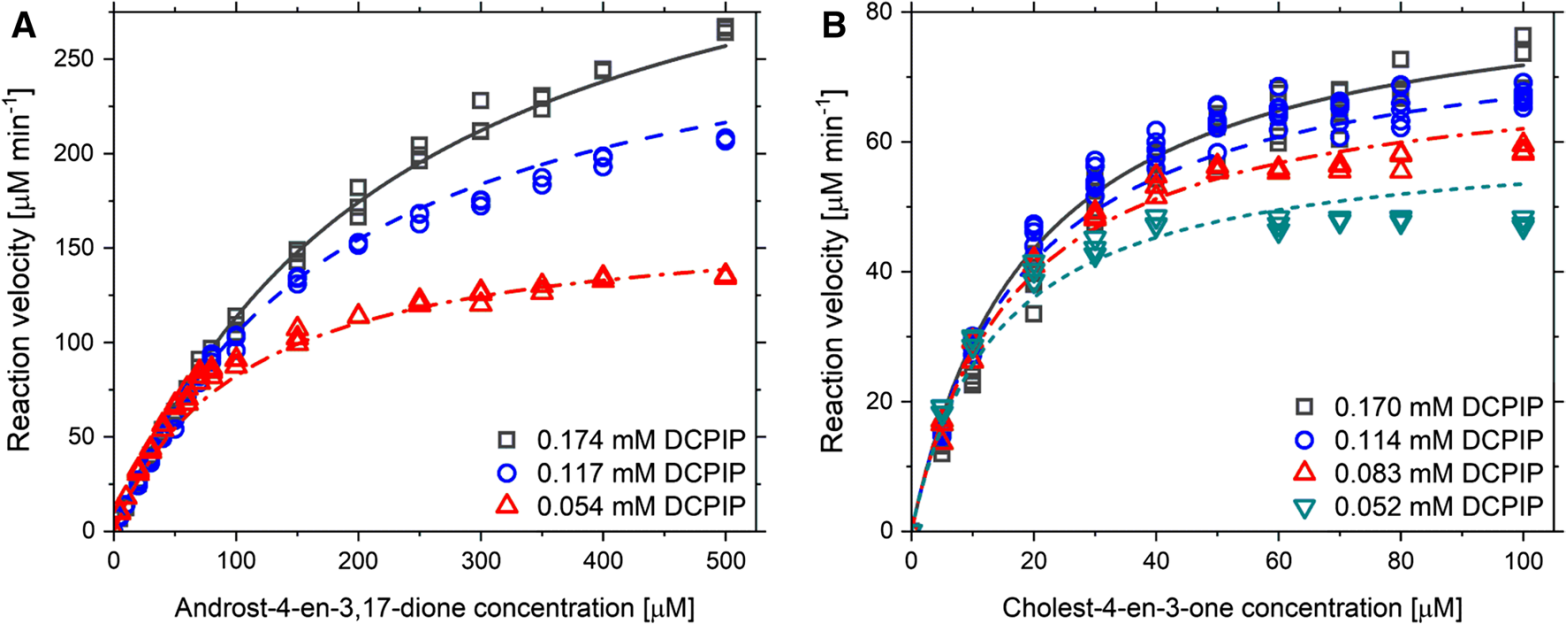
Results of steady-state kinetics for dehydrogenation reaction of androst-4-en-3,17-dione **A** and cholest-4-en-3-one **B** catalyzed by AcmB

To determine the substrate specificity of AcmB we conducted steady-state kinetic studies for androst-4-en-3,17-dione and cholest-4-en-3-one. 2,6-Dichloroindphenol was used as an artificial electron acceptor. Since the solubility of cholest-4-en-3-one in aqueous solutions is close to zero, measurements were carried out with 2% HBC and 2% EGME. The reaction velocities were presented in Fig. 4. The Ping-Pong bi bi (non-sequential) mechanism was fitted to the obtained data (4) [46].

4 $$\mathrm{v}=\frac{{\mathrm{V}}_{\mathrm{m}\mathrm{a}\mathrm{x}}{\left[\mathrm{S}\right]}_{\mathrm{t}}[\mathrm{D}\mathrm{C}\mathrm{P}\mathrm{I}\mathrm{P}]}{{\mathrm{K}}_{\mathrm{m}\mathrm{S}}\left[\mathrm{D}\mathrm{C}\mathrm{P}\mathrm{I}\mathrm{P}\right]+{\mathrm{K}}_{\mathrm{m}\mathrm{D}\mathrm{C}\mathrm{P}\mathrm{I}\mathrm{P}}{\left[\mathrm{S}\right]}_{\mathrm{t}}+{\left[\mathrm{S}\right]}_{\mathrm{t}}[\mathrm{D}\mathrm{C}\mathrm{P}\mathrm{I}\mathrm{P}]}$$

Due to the presence of HBC in the system, the substrate was available to the enzyme mostly in the form of 1:1 and/or 1:2 complexes (99% for S(HBC) AD complex, and 53.52 and 43.55% for S(HBC) and S(HBC)_2_ complexes with cholest-4-en-3-one, see Additional file 1: Table S2 for details). As a result, only in the case of AD, we could assume that the observed kinetics was mostly coming from the S(HBC) form of the substrate while in the case of cholest-4-en-3-one the kinetics of E:S complex formation was much more complicated and proceeds according to Eq. (5).
5E – enzyme (KSTD); E’ – enzyme in its reduced form; S – substrate 1 (steroid); HBC – 2-hydroxypropyl-β-cyclodextrin and DCPIP – substrate 2 (artificial electron acceptor).

Although it is possible to derive the kinetic equation that takes into account all forms of the substrate present in the solution, the correct fitting of all constants requires collecting kinetic data for different concentrations of HBC, steroid and DCPIP. Our experimental data turned out to be significantly underdetermined to handle such a complex non-linear fit. However, as the main purpose of our study was to compare different 3-ketosteroid dehydrogenases under the same conditions (here concentration of HBC which determines the distribution of S:HBC complexes) we have decided to present the kinetic parameters in the function of the total substrate concentration. Due to that, kinetic curves were fitted to the concentration of the total substrate.

Under these conditions, the maximum velocity of reaction for AcmB (V_max_) was over 8 times higher for AD than for cholest-4-en-3-one. However, AcmB affinity (K_mS_) to cholest-4-en-3-one turned out to be over 20**-**times higher compared with AD. As a result, the enzyme catalytic efficiencies (k_cat_/K_mS_) for cholest-4-en-3-one was more than two-fold higher than that determined for AD (Table 3).

**Table 3:** Apparent kinetic parameters of the purified AcmB

Substrate	K_mS_ [μM]	K_mDCPIP_ [μM]	V_max_ [μM min^−1^]	k_cat_ [s^−1^]	k_cat_/K_mS_ [M^−1^ s^−1^]
Androst-4-en-3,17-dione (**1**)	529.2 ± 32.5	223.5 ± 16.9	859.2 ± 45.5	46.1 ± 2.3	(0.87 ± 0.05)∙10^5^
Cholest-4-en-3-one (**2**)	23.7 ± 1.16	37.3 ± 3.3	104.6 ± 2.7	5.8 ± 0.2	(2.45 ± 0.01)∙10^5^

It should be noted here that DCPIP is also complexed by HBC. This fact however does not impact the comparisons of K_mS_ values, as experiments were conducted for both substrates with the same type and concentrations of oxidation agent (DCPIP) and the HBC concentration was 30 to 84-times higher than DCPIP concentration. The influence of DCPIP complexation (such as decrease of HBC concentration) on obtained kinetic parameters was negligible.

In the genome of *S. denitrificans* we have identified two other putative AcmB homologs, which biological role has not been yet established. For this study, we selected gene encoding “3-oxosteroid 1-dehydrogenase” (GenBank: SMB21450.1) which we named AcmB2 due to its high sequence similarity to AcmB (44% identity and 62% similarity). The recombinant enzyme was expressed in *E. coli* BL21(DE3)Magic and purified on Ni–NTA matrix. To check if AcmB2 also exhibited activity with C17-substituted steroids we performed mini reactor tests with 100 μM of AD, cholest-4-en-3-one or diosgenone with the addition of 2% HBC (Additional file 1: Figs. S1–S8). In a reactor with diosgenone, we introduced a fivefold higher concentration of AcmB2 than for other mini reactors to account for the observed lower rate of the initial tests. After 16 h of reaction, we observed significant conversions of all substrates i.e., 77.2 ± 1.5% for AD, 80.2 ± 2.7% for cholest-4-en-3-one and 53 ± 3.5% for diosgenone. These results show that other 3-ketosteroid dehydrogenase from *S. denitrifican*s exhibits similar substrate specificity as AcmB.

### KSTD1 substrate spectrum

**Fig. 5:**
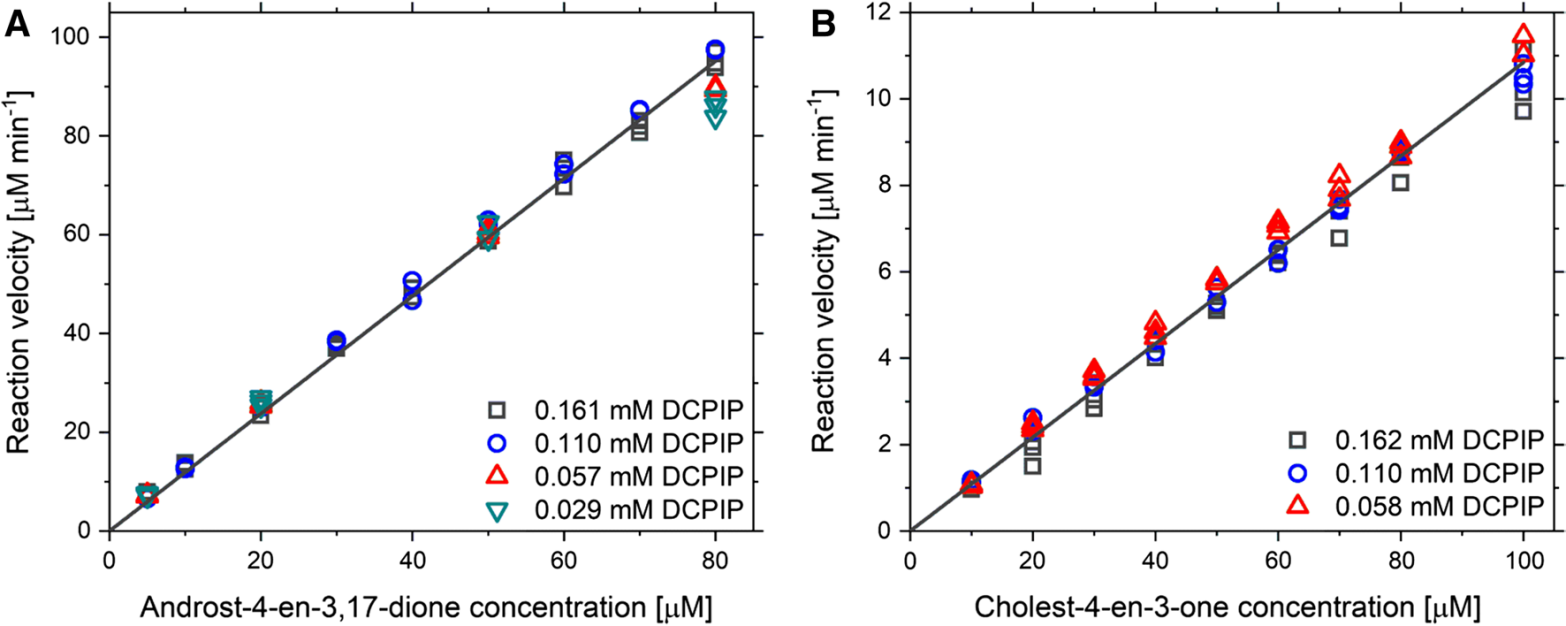
Results of steady-state kinetics for dehydrogenation reaction of androst-4-en-3,17-dione **A** and cholest-4-en-3-one **B** catalyzed by KSTD1

The kinetic measurements for KSTD1 were performed at the same conditions as described previously for AcmB, except for the pH of the buffer solution. Surprisingly, the affinity of KSTD1 from *R. erythropolis* to AD and cholest-4-en-3-one turned out to be significantly lower than this exhibited by AcmB and it was not possible to reach enzyme saturation conditions in the presence of HBC. The solubility of steroids was limited even with solubilizing additives. On the other hand, a further increase of the HBC concentration results in an apparent K_m_ shift toward higher values as a consequence of substrate sequestration (i.e., decrease of free substrate concentration and stabilization of the substrate in S(HBC)_n_ form). Nevertheless, we have decided to estimate the first-order rate coefficients (V_max_/K_m_) for the low substrate concentrations. The linear function was fitted to the reaction velocities determined for 5 – 100 μM steroids concentrations (Fig. 5). To obtain comparable kinetic parameters, the same procedure was applied to AcmB (Additional file 1: Fig. S9).

For the small concentrations of the first substrate (S <  < K_m_) the velocity equation becomes invariant to the concentration of the second substrate (DCPIP) and simplifies to a first-order linear equation:

6 $$\mathrm{v}=\frac{{\mathrm{V}}_{\mathrm{m}\mathrm{a}\mathrm{x}}[\mathrm{S}]}{{\mathrm{K}}_{\mathrm{m}}}$$

The apparent catalytic efficiency k_cat_/K_m_ of AcmB and KSTD1 for AD was of the same order. KSTD1 proved to be a slightly (22%) better catalyst for the AD Δ^1^-dehydrogenation than AcmB. In the case of the reaction with cholest-4-en-3-one, the k_cat_/K_m_ value measured for AcmB was almost 20-times higher than for KSTD1. The KSTD1 was 10-times better in the Δ^1^-dehydrogenation of AD vs cholest-4-en-3-one (Table 4).

**Table 4:** Apparent kinetic parameters of the purified AcmB and KSTD1

Substrate	AcmB	KSTD1
	k_cat_/K_m_ [M^−1^ s^−1^]	k_cat_/K_m_ [M^−1^ s^−1^]
Androst-4-en-3,17-dione (**1**)	(4.00 ± 0.04)∙10^6^	(5.17 ± 0.03)∙10^6^
Cholest-4-en-3-one (**2**)	(9.25 ± 0.17)∙10^6^	(4.71 ± 0.03)∙10^5^

The obtained results confirmed undeniably the ability of KSTD1 to catalyze Δ^1^-dehydrogenation of steroids with the aliphatic chain on the C17 position. Due to the low cholest-4-en-3-one bioavailability and the lower KSTD1 affinity to this steroid, it was not possible to determine the actual KSTD1 substrate spectrum without the addition of solubilizing agent [29].

Based on the results of MD simulations for KSTD1 and diosgenone ES complex and the experimentally confirmed activity of AcmB and AcmB2 with diosgenon [8] we decided to investigate if KSTD1 is also able to catalyze the reaction with 6-member ring steroids. The mini reactor tests proved that in presence of 2% HBC 100 μM diosgenone can be converted with excellent 97.3 ± 0.8% conversion in 20 min.

## Discussion

The results of theoretical MD simulations for 3-ketosteroid Δ^1^-dehydrogenase from *R. erythropolis* SQ1 have shown that neither isooctyl side chain of cholest-4-en-3-one nor polycyclic substituent of diosgenone is a steric hindrance for the reaction catalyzed by KSTD1. Only the gonane ring is recognized by KSTD active site and the additional substituents or rings are protruding into the solvent, outside of the enzyme. This hypothesis was successfully confirmed by experimental kinetic studies and reactor tests. Nevertheless, the affinity and the catalytic efficiency of KSTD1 from *R. erythropolis* for cholest-4-en-3-one turned out to be significantly lower than that determined for AcmB from *S. denitrificans*. Additionally, a more hydrophobic character of the AcmB surface when compared to KSTD1 may also contribute to its higher affinity to more hydrophobic substrates [47].

Our results are in line with reports of Wang et al*.* [22], who showed that KSTD from *R. erythropolis* WY 1406 strain can also convert substrates with extended C17 substituent, like 21-acetoxy-pregna-4,9(11),16-triene-3,20-dione. In addition to AcmB and the new AcmB2 [8, 21], there were some literature reports about KSTDs activity towards steroids with a more complex side chain on the C17 position [18–20, 23, 48]. Zhang et al. described five KSTD isoenzymes from *Gordonia neofelifaecis* of which four exhibited activity toward cholest-4-en-3-one. Importantly, the authors conducted experiments with 0.05% Tween 80 acting as a solubilizing agent [20].

Poor solubility of steroids with the aliphatic or polycyclic substituents at the C17 atom results in their low bioavailability to the enzyme. Compounded with a low affinity of most KSTDs to more complex steroids, these two factors may lead to inaccurate conclusions regarding substrate specificity. The results obtained for KSTD1, AcmB, AcmB2, as well as for KSTDs from *G. neofelifaecis* [20] indicate that the use of cyclodextrins or detergents can influence enzymes substrate specificity analysis. Such analysis, however, requires determination of the HBC/steroid stability constants which in turn help in the determination of the real concentration of the substrate, but complicate the observed kinetics due to different ΔGs of E:S formation for S, S(HBC) or S(HBC)_2_ forms of the substrate. Furthermore, it should be underlined here that there are several different methods for the determination of these constants (HPLC, NMR, FT-IR, XRD or calorimetry-based, etc.) and they usually differ in the delivered values [49, 50].

Despite these difficulties, the application of solubilizing agent seems indispensable for the determination of the active substrates, especially when KSTD affinity to the compounds with an aliphatic chain on the C17 position is high, as in AcmB (apparent K_mS_ of 23.7 µM). Our results indicate that AD may not be a native substrate of AcmB. The established affinity toward AD is, however, the affinity toward S(HBC) complex (apparent K_mS_ of 529.2 µM) as we know from our previous kinetic studies that K_m_ value measured in the assay without HBC was an order of magnitude lower (apparent K_m_ = 59.6 ± 3.0 µM at 200 µM DCPIP and 30 °C) [8] than in the assay with HBC (Tables 3 and 4). The K_mS_ determined in this work for cholest-4-en-3-one is lower than those observed for AD without HBC. Furthermore, as 97% of the cholest-4-en-3-one was in the form of complexes with HBC we can assume that the real K_mS_ for cholest-4-en-3-one (if it was possible to measure it without HBC) is even lower.

The difference in the affinity of KSTDs to C17-substituted steroids might result from their structural differences. The homology modeling of KSTD from *Arthrobacter simplex* (active towards cholest-4-en-3-one) showed the presence of an additional loop close to the enzyme active site, non-observed in the crystal structure of KSTD1 form *R. erythropolis* [34, 51]. Interestingly, the same feature was noticed in the AcmB homology model [52].

Our findings have implications for the understanding of the physiological role of AcmB. Initial studies on the anoxic cholesterol metabolism of *S. denitrificans* reported new metabolites, cholest-1,4-diene-3-one and 25-hydroxycholest-1,4-dien-3-one, and suggested that the double bond between the C1 and C2 atoms have had to be introduced at the early stage of cholesterol mineralization. As a result, cholest-4-en-3-one was considered as the AcmB native substrate. [25, 26]. However, subsequent kinetic studies demonstrated the AcmB preference for C19 – C21 steroids [21], which was supported by the localization of the enzyme on the cytoplasmic side of the bacterial inner membrane [27]. Due to that fact, Lin et al*.* deduced that the AcmB native substrate is rather the AD [27]. Kinetic studies indicating AcmB localization were estimated based on the enzyme activity measured in particular fractions, not by e.g., antibody technique and AcmB specific activity was calculated for the total protein extracted from the membrane. Importantly, the KSTD activity was still detected significantly in the peripheral-periplasmic fraction [27]. In this work, we demonstrated, that a study of the enzyme–substrate specificity and affinity in the case of hydrophobic steroids without the solubilizing agent addition may lead to misinterpretation of achieved results. In the presence of HBC, the AcmB affinity toward cholest-4-en-3-one turned out to be significantly higher than for AD (even taking into consideration all complications introduced by substrate complexation by HBC).

Finally, one has to remember about two other AcmB sequence homologs, from which we confirmed 3-ketosteroid Δ^1^-dehydrogenase activity for one of them, AcmB2. Therefore it may well be that *S. denitrificans* deploys different KSTD enzymes on both sides of its inner membrane, which specialize in converting different types of 3-ketosteroids (as reported already for KSTD isoenzymes from *R. erythopolis* [53, 54]). This aspect definitely requires further studies.

## Conclusions

Our results indicate that it is necessary to test KSTD substrates in the presence of solubilizing agents such as HBC, to discern truly inactive steroids from those which under experimental conditions are in too low concentrations. Unfortunately, the presence of HBC introduces additional complications to the kinetic model due to the presence of different forms of the substrate (S and S(HBC)_n_). Our modeling studies suggest that in principle all KSTDs, which share structural characteristics with KSTD1 should be able to convert more hydrophobic substrates with extended ring system or C17 substituent. This conclusion has an important impact on the biotechnological application of those enzymes.

## Supplementary Information

**Additional file 1.** **Fig. S1.** Root Mean Square Deviation and selected distances during MD simulation for androst-4-en-3,17-dione. Fragment of trajectory selected for MMPBSA calculations is marked with red rectangles. **Fig. S2.** Root Mean Square Deviation and selected distances during MD simulation for cholest-4-en-3-one. Fragment of trajectory selected for MMPBSA calculations is marked with red rectangles. **Fig. S3.** Root Mean Square Deviation and selected distances during MD simulation for diosgenone. Fragment of trajectory selected for MMPBSA calculations is marked with red rectangles. **Fig. S4.** Root Mean Square Deviation and selected distances during MD simulation for androstanolone. Fragment of trajectory selected for MMPBSA calculations is marked with red rectangles. **Fig. S5.** Root Mean Square Deviation (and selected distances during MD simulation for progesterone. Fragment of trajectory selected for MMPBSA calculations is marked with red rectangles. **Fig. S6.** Root Mean Square Deviation (and selected distances during MD simulation for testosterone propionate. Fragment of trajectory selected for MMPBSA calculations is marked with red rectangles. **Fig. S7.** Root Mean Square Deviation and selected distances during MD simulation for 6-dehydrotestosterone acetate. Fragment of trajectory selected for MMPBSA calculations is marked with red rectangles. **Table S1.** Fit parameters and statistics of phase solubility diagrams of steroids in the solution of HBC and 2% EGME. **Table S2.** Percentage of cholest-4-en-3-one forms dependent on initial substrate concentration; S: cholest-4-en-3-one, HBC: 2-hydroxypropyl-β-cyclodextrin. **Table S3.** Fit statistics of the AcmB Ping-Pong bi bi (non-sequential) mechanism. **Fig. S8.** Progress of the 1,2-dehydrogenation of 0.1 mM AD **A** and cholest-4-en-3-one **B** with 6.55 nM AcmB2 and 0.1 mM diosgenone **C** with 65.5 nM AcmB2 in the presence of 0.15 mM DCPIP in 50 mM Tris-HCl buffer pH 8.0. S–substrate; P–product. **Fig. S9.** Results of steady-state kinetics for dehydrogenation reaction of androst-4-en-3.17-dione **A** and cholest-4-en-3-one **B** calatyzed by AcmB. Reaction velocities were measured in 0.1 M K_2_HPO_4_/KH_2_PO_4_ buffer pH 6.5 with 2% HBC. 2% EGME. 0.052–0.174 mM DCPIP. 5–80 μM steroids and 0.30 μM of AcmB at 30 °C.**Table S4.** Fit parameters and statistics of AcmB and KSTD1 steady-state kinetics results.

## Data Availability

All data generated and analyzed during this study are included in this published article and its Additional file 1. Additional raw data (MD trajectories, raw kinetic data) are available on request.

## Abbreviations

AcmB: 3-Ketosteroid Δ^1^-dehydrogenase 1 from *Sterolibacterium denitrificans*
AcmB2: 3-Ketosteroid Δ^1^-dehydrogenase 2 from *Sterolibacterium denitrificans*
ACN: Acetonitrile
AD: Androst-4-en-3,17-dione
AMBER: Assisted model building with energy refinement
ANB: 1,4-Androstadien-3,71-dione
BME: β-Mercaptoethanol
DAD: Diode array detector
DCPIP: 2,6-Dichloroindophenol
dCTP: Deoxycytidine triphosphate
dGTP: Deoxyguanosine triphosphate
DMSO: Dimethyl sulfoxide
DTT: Dithiothreitol
EGME: 2-Methoxyethanol
ESP: Electrostatic Potential
FAD: Flavin adenine dinucleotide
HBC: 2-Hydroxypropyl-β-cyclodextrin
HPLC: High performance liquid chromatography
IPTG: Isopropyl β-D-1-thiogalactopyranoside
kbp: Kilobase pare
k_cat_: Catalytic constant/turnover number
K_m_: Michaelis constant
K_S_: HBC/steroid complex stability constant
KSTD(s): 3-Ketosteroid Δ^1^-dehydrogenase(s)
KSTD1: 3-Ketosteroid Δ^1^-dehydrogenase from *R. erythropolis* SQ1
LB: Lennox Broth
MD: Molecular dynamics
MM-PBSA: Molecular mechanics energies combined with the Poisson–Boltzmann and surface area continuum solvation
MS: Mass spectrometry
NPT: Isothermal-isobaric ensemble
NVT: Canonical ensemble
PDB: Protein Data Bank
PMSF: Phenylmethylsulfonyl fluoride
RESP: Restrained Electrostatic Potential
V_max_: Maximal reaction velocity
