# Supplementary material for: Universal capability of 3-ketosteroid Δ1-dehydrogenases to catalyze Δ1-dehydrogenation of C17-substituted steroids
Source: Microb Cell Fact. 2021 Jun 23;20:119. doi: 10.1186/s12934-021-01611-5 (PMC8220720; doi:10.1186/s12934-021-01611-5)
Supplement: Supplementary file 1 — Additional file 1. Fig. S1. Root Mean Square Deviation and selected distances during MD simulation for androst-4-en-3,17-dione. Fragment of trajectory selected for MMPBSA calculations is marked with red rectangles. Fig. S2. Root Mean Square Deviation and selected distances during MD simulation for cholest-4-en-3-one. Fragment of trajectory selected for MMPBSA calculations is marked with red rectangles. Fig. S3. Root Mean Square Deviation and selected distances during MD simulation for diosgenone. Fragment of trajectory selected for MMPBSA calculations is marked with red rectangles. Fig. S4. Root Mean Square Deviation and selected distances during MD simulation for androstanolone. Fragment of trajectory selected for MMPBSA calculations is marked with red rectangles. Fig. S5. Root Mean Square Deviation (and selected distances during MD simulation for progesterone. Fragment of trajectory selected for MMPBSA calculations is marked with red rectangles. Fig. S6. Root Mean Square Deviation (and selected distances during MD simulation for testosterone propionate. Fragment of trajectory selected for MMPBSA calculations is marked with red rectangles. Fig. S7. Root Mean Square Deviation and selected distances during MD simulation for 6-dehydrotestosterone acetate. Fragment of trajectory selected for MMPBSA calculations is marked with red rectangles. Table S1. Fit parameters and statistics of phase solubility diagrams of steroids in the solution of HBC and 2% EGME. Table S2. Percentage of cholest-4-en-3-one forms dependent on initial substrate concentration; S: cholest-4-en-3-one, HBC: 2-hydroxypropyl-β-cyclodextrin. Table S3. Fit statistics of the AcmB Ping-Pong bi bi (non-sequential) mechanism. Fig. S8. Progress of the 1,2-dehydrogenation of 0.1 mM AD A and cholest-4-en-3-one B with 6.55 nM AcmB2 and 0.1 mM diosgenone C with 65.5 nM AcmB2 in the presence of 0.15 mM DCPIP in 50 mM Tris-HCl buffer pH 8.0. S–substrate; P–product. Fig. S9. Results of steady-state kinetics for deh [file 12934_2021_1611_MOESM1_ESM.docx]

**Supporting Information**

**Universal capability of 3-ketosteroid Δ^1^-dehydrogenases to catalyze Δ^1^-dehydrogenation of C17- substituted steroids**

Patrycja Wójcik^1^, Michał Glanowski^1^, Agnieszka M. Wojtkiewicz^1^, Ali Rohman^2,3,4^, Maciej Szaleniec^1^*

*Correspondence: maciej.szaleniec@ikifp.edu.pl

^1^ Jerzy Haber Institute of Catalysis and Surface Chemistry Polish Academy of Sciences, Niezapominajek 8, PL30239 Krakow, Poland

^2^ Department of Chemistry, Faculty of Science and Technology, Universitas Airlangga, Surabaya 60115, Indonesia

^3^ Laboratory of Proteomics, Research Center for Bio-Molecule Engineering (BIOME), Universitas Airlangga, Surabaya 60115, Indonesia

^4^ Laboratory of Biophysical Chemistry, University of Groningen, 9747 AG Groningen, The Netherlands

**Results**

**Theoretical prediction of substrate specificity**

**
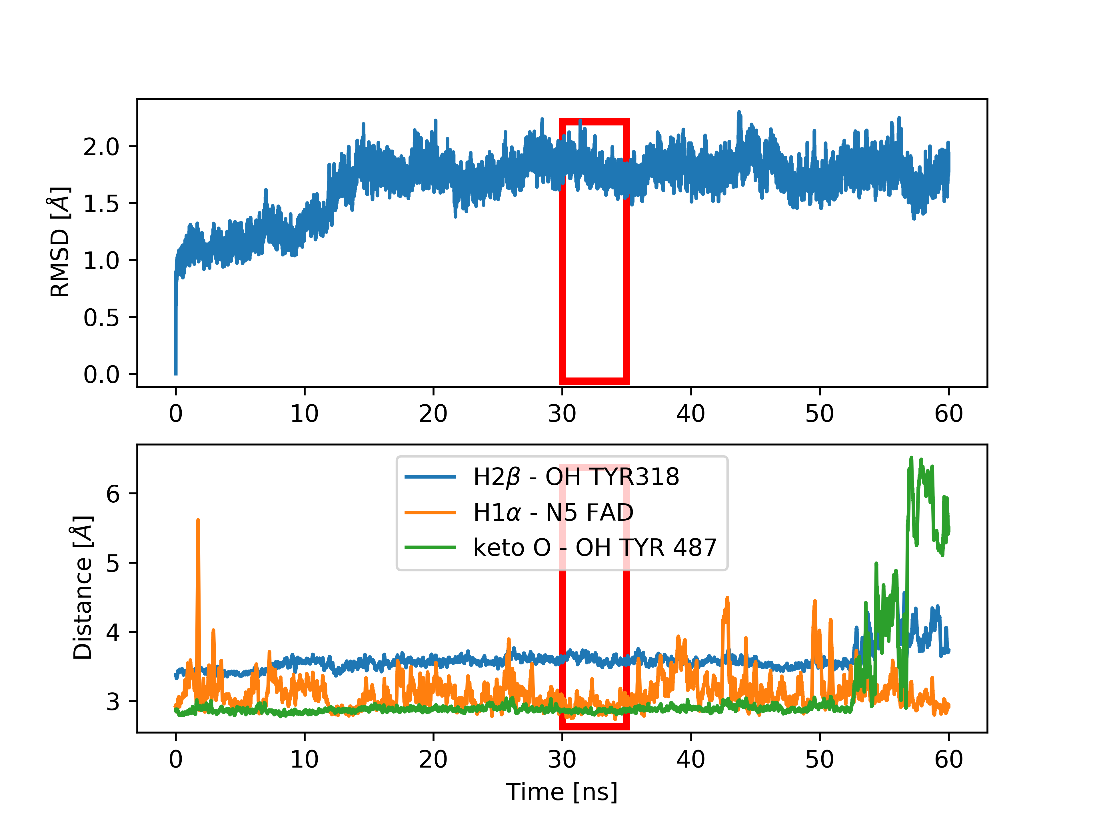
**

**Fig. S1** Root Mean Square Deviation (top graph) and selected distances (bottom graph, smoothed by moving average) during MD simulation for androst-4-en-3,17-dione. Fragment of trajectory selected for MMPBSA calculations is marked with red rectangles.

**
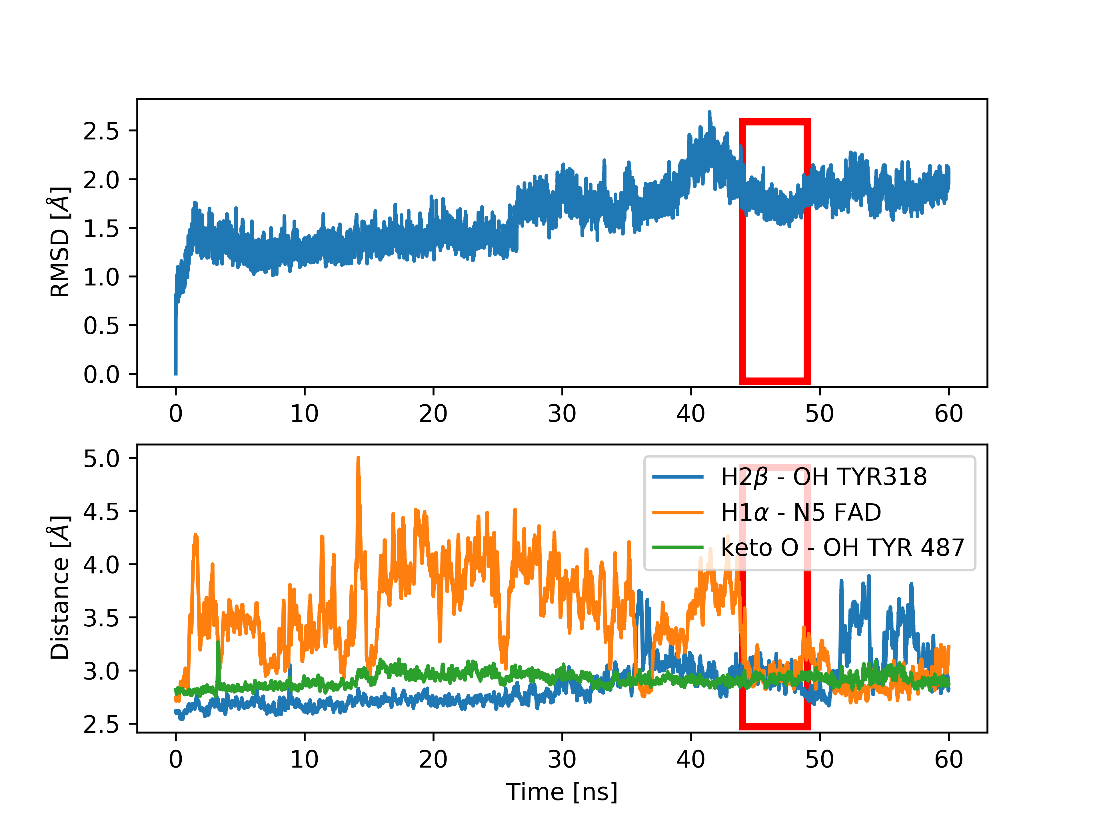
**

**Fig. S2** Root Mean Square Deviation (top graph) and selected distances (bottom graph, smoothed by moving average) during MD simulation for cholest-4-en-3-one. Fragment of trajectory selected for MMPBSA calculations is marked with red rectangles.

**
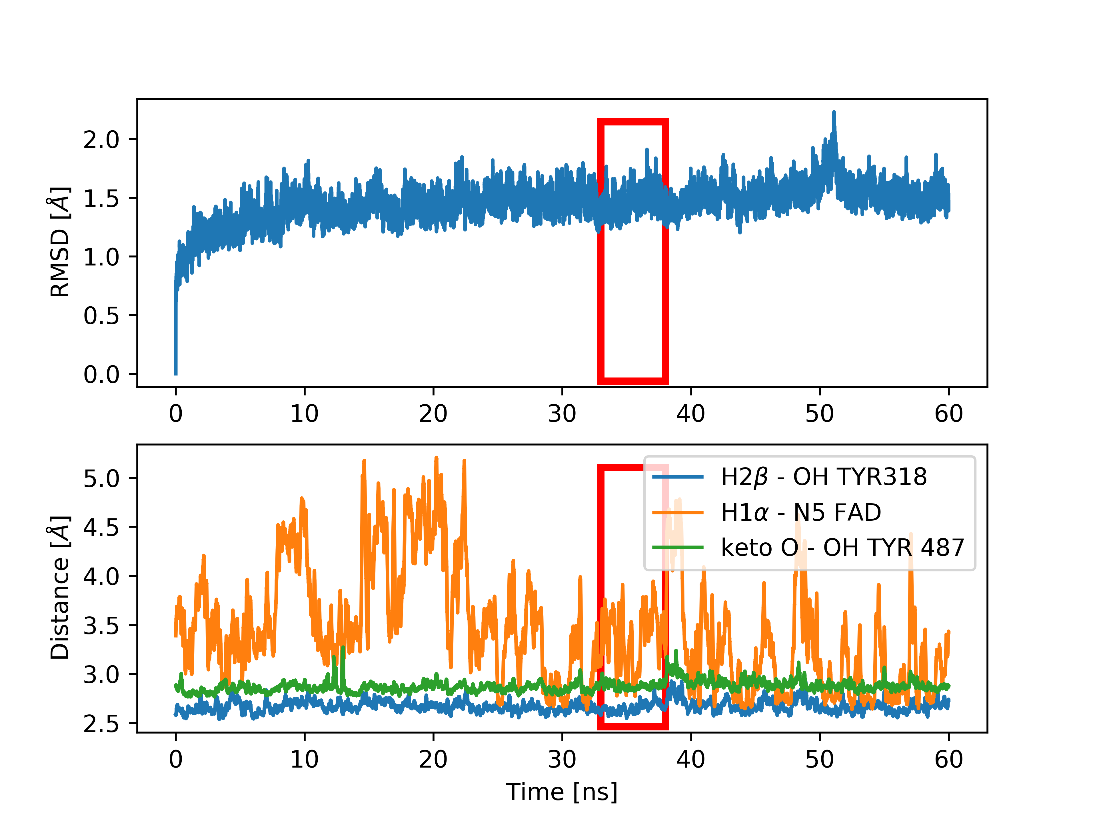
**

**Fig. S3** Root Mean Square Deviation (top graph) and selected distances (bottom graph, smoothed by moving average) during MD simulation for diosgenone. Fragment of trajectory selected for MMPBSA calculations is marked with red rectangles.

**
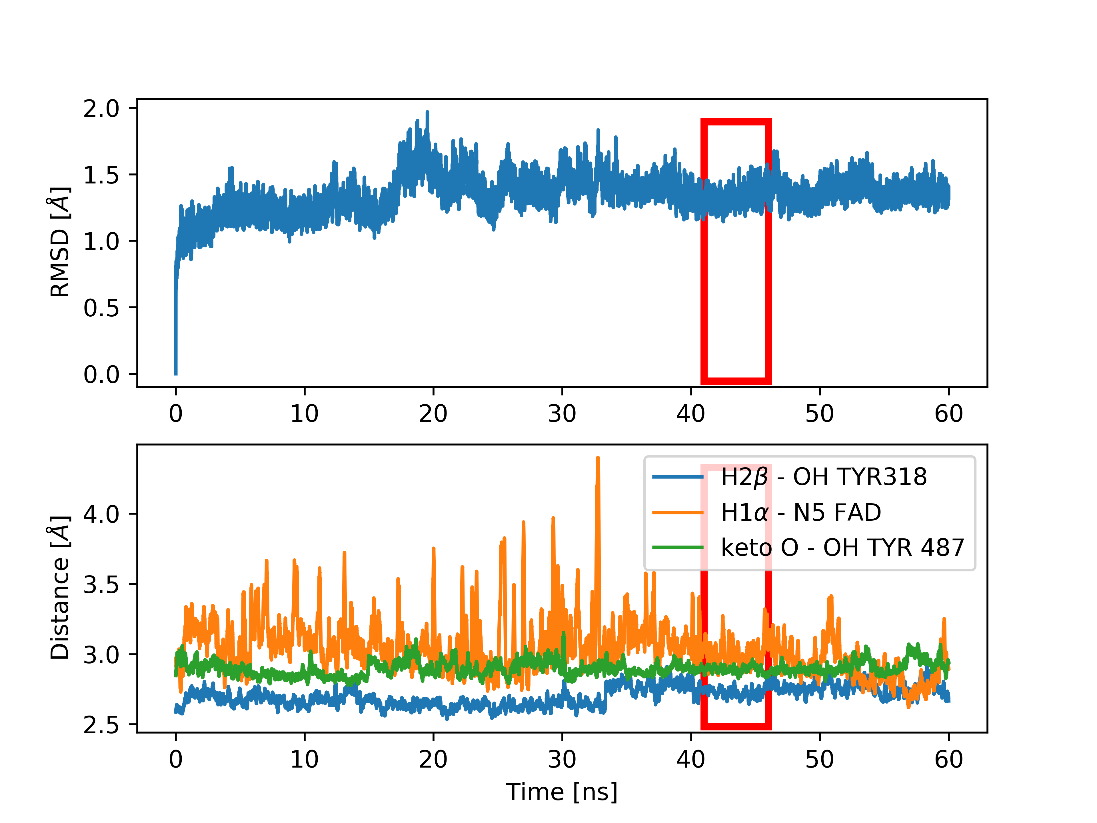
**

**Fig. S4** Root Mean Square Deviation (top graph) and selected distances (bottom graph, smoothed by moving average) during MD simulation for androstanolone. Fragment of trajectory selected for MMPBSA calculations is marked with red rectangles.

**
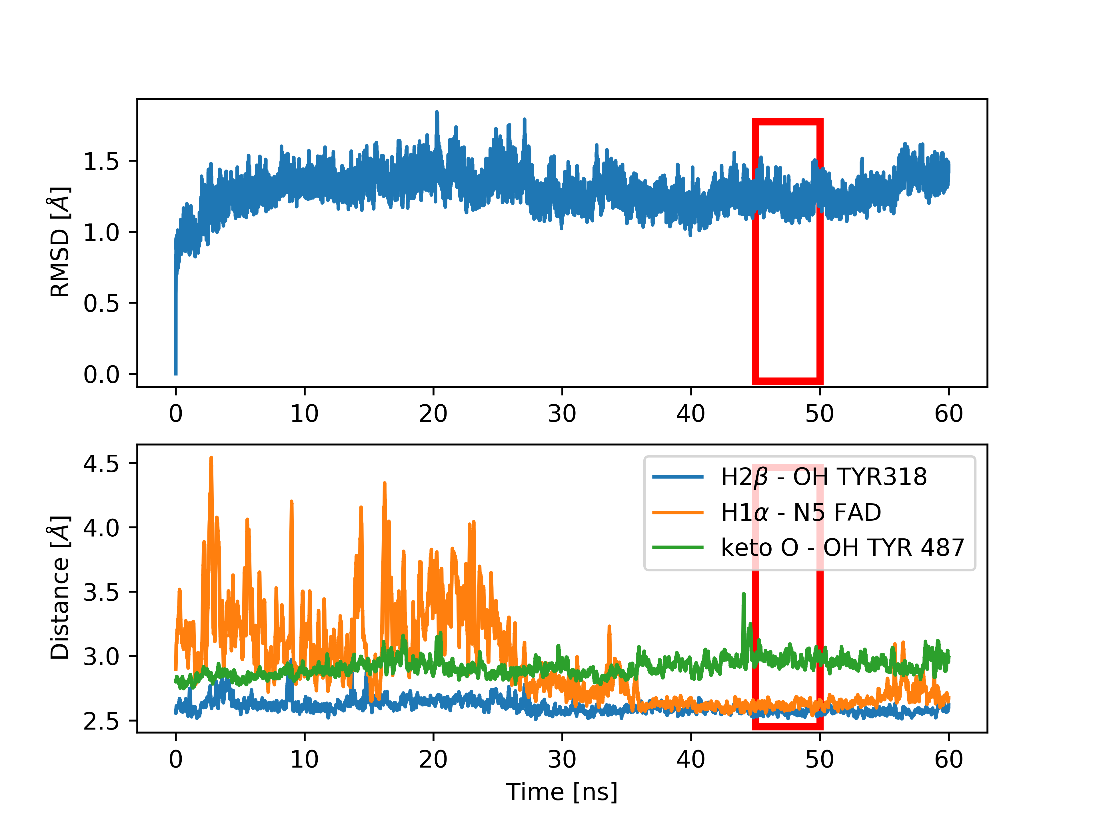
**

**Fig. S5** Root Mean Square Deviation (top graph) and selected distances (bottom graph, smoothed by moving average) during MD simulation for progesterone. Fragment of trajectory selected for MMPBSA calculations is marked with red rectangles.

**
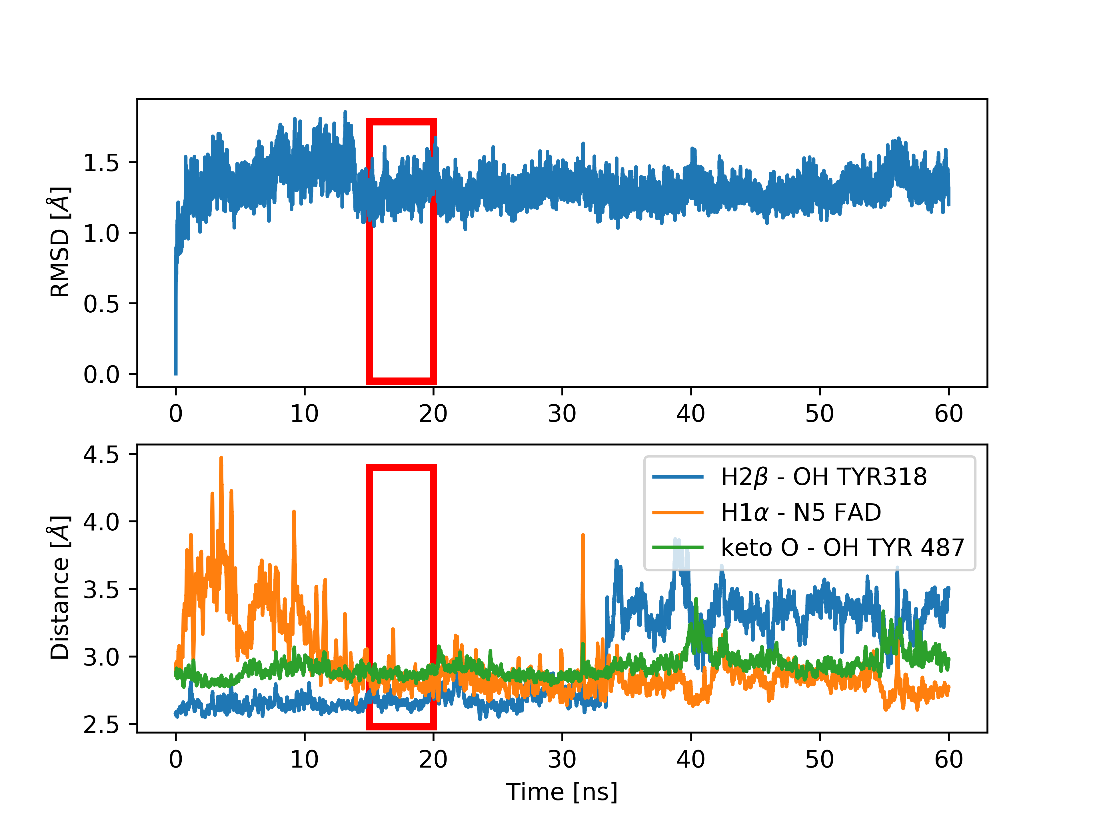
**

**Fig. S6** Root Mean Square Deviation (top graph) and selected distances (bottom graph, smoothed by moving average) during MD simulation for testosterone propionate. Fragment of trajectory selected for MMPBSA calculations is marked with red rectangles.


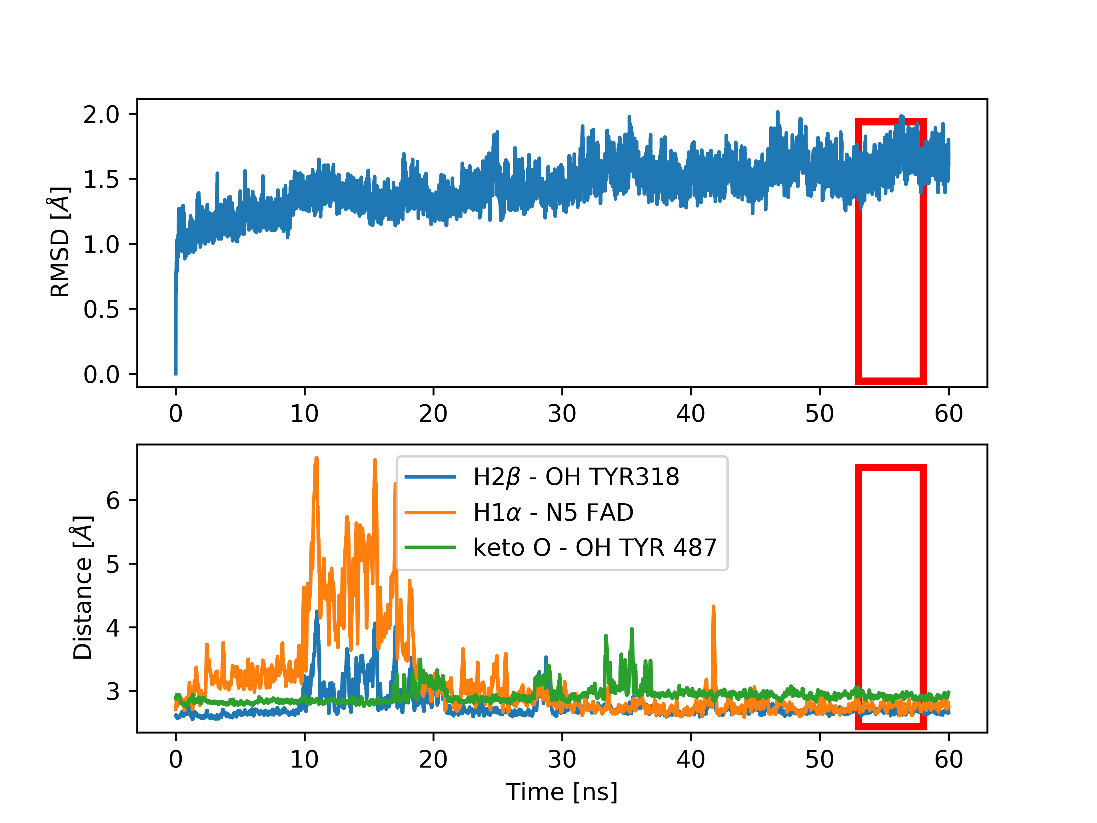


**Fig. S7** Root Mean Square Deviation (top graph) and selected distances (bottom graph, smoothed by moving average) during MD simulation for 6-dehydrotestosterone acetate. Fragment of trajectory selected for MMPBSA calculations is marked with red rectangles.

**HBC/steroid inclusion complex formation**

**Table S1 Fit parameters and statistics of phase solubility diagrams of steroids in the solution of HBC and 2% EGME at 30°C**

|  | **Substrate** | |
| --- | --- | --- |
| **Parameter** | Androst-4-en-3,17-dione (**1**) | Cholest-4-en-3-one (**2**) |
| Equation | y = ax + b | y = ax^2^ + bx + c |
| a | 0.52661 ± 0.00859 | 0.00035 ± 0.00001 |
| b | 0.14420 ± 0.00714* | 0.00611 ± 0.00081 |
| c | - | 0.00478 ± 0.00054* |
| R^2^ | 0.99869 | 0.9989 |
| Adj. R^2^ | 0.99843 | 0.99835 |

* measured values

**Table S2 Percentage of cholest-4-en-3-one forms dependent on initial substrate concentration; S - cholest-4-en-3-one, HBC – 2-hydroxypropyl-β-cyclodextrin**

| **[S]_t_ [μM]** | **% [S]** | **% [S(HBC)]** | **% [S(HBC)_2_]** |
| --- | --- | --- | --- |
| 5 | 2.87 | 53.21 | 43.93 |
| 10 | 2.87 | 53.22 | 43.91 |
| 20 | 2.87 | 53.24 | 43.89 |
| 30 | 2.88 | 53.26 | 43.86 |
| 40 | 2.88 | 53.28 | 43.84 |
| 50 | 2.88 | 53.30 | 43.81 |
| 60 | 2.89 | 53.32 | 43.79 |
| 70 | 2.89 | 53.35 | 43.76 |
| 80 | 2.90 | 53.37 | 43.74 |
| 100 | 2.90 | 53.41 | 43.69 |

**AcmBs native substrate**

**Table S3 Fit statistics of the AcmB Ping-Pong bi bi (non-sequential) mechanism**

|  | **Substrate** | |
| --- | --- | --- |
| **Parameter** | Androst-4-en-3.17-dione (**1**) | Cholest-4-en-3-one (**2**) |
| Reduced χ^2^ | 32.77842 | 11.68547 |
| R^2^ | 0.99318 | 0.9749 |
| Adj. R^2^ | 0.99309 | 0.97464 |
| AICc | 546.15167 | 484.35427 |

**
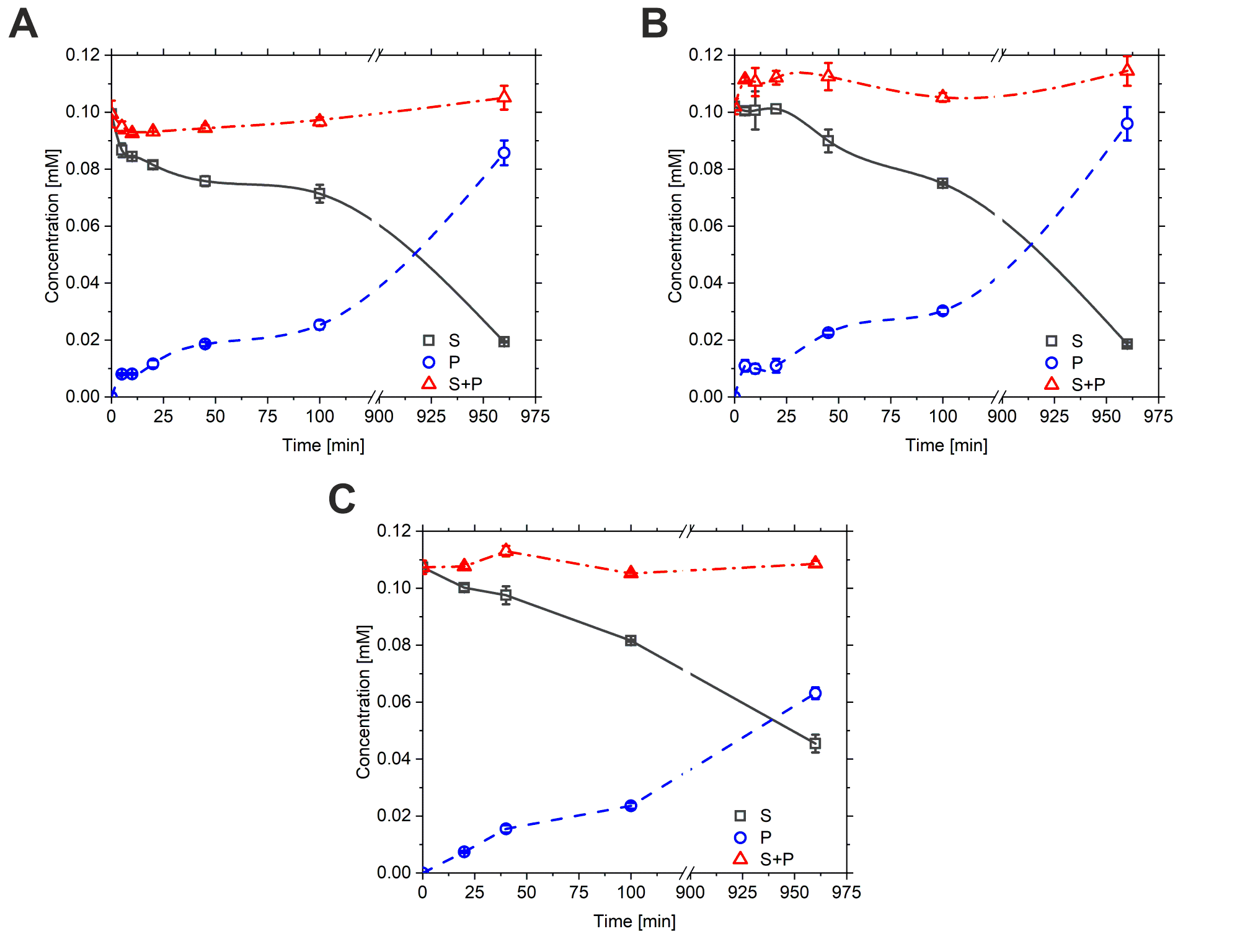
**

**Fig. S8** Progress of the 1.2-dehydrogenation of 0.1 mM AD (**A**) and cholest-4-en-3-one (**B**) with 6.55 nM AcmB2 and 0.1 mM diosgenone (**C**) with 65.5 nM AcmB2 in the presence of 0.15 mM DCPIP in 50 mM Thris-HCl buffer pH 8.0. S – substrate; P – product.

**KSTD1 substrate spectrum**

**
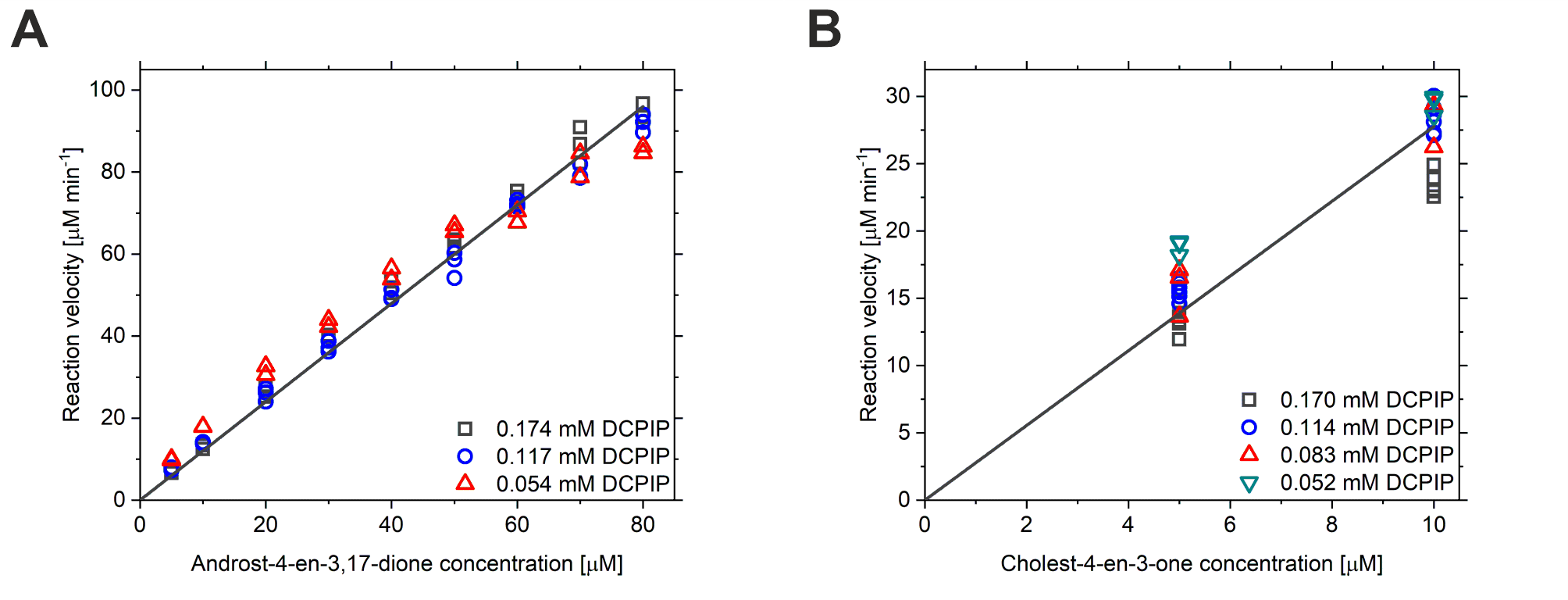
**

**Fig. S9** Results of steady-state kinetics for dehydrogenation reaction of androst-4-en-3.17-dione (**A**) and cholest-4-en-3-one (**B**) calatyzed by AcmB. Reaction velocities were measured in 0.1 M K_2_HPO_4_/KH_2_PO_4_ buffer pH 6.5 with 2% HBC. 2% EGME. 0.052 – 0.174 mM DCPIP. 5 – 80 μM steroids and 0.30 μM of AcmB at 30°C.

**Table S4 Fit parameters and statistics of AcmB and KSTD1 steady-state kinetics results**

|  |  | **Substrate** | |
| --- | --- | --- | --- |
|  | **Parameter** | Androst-4-en-3.17-dione (**1**) | Cholest-4-en-3-one (**2**) |
| **KSTD1** | Equation | y = ax + b | y = ax + b |
|  | a | 1.18924 ± 0.00672 | 0.10844 ± 0.000668728 |
|  | b | 0 | 0 |
|  | R^2^ | 0.99755 | 0.99704 |
|  | Adj. R^2^ | 0.99751 | 0.997 |
| **AcmB** | Equation | y = ax + b | y = ax + b |
|  | a | 1.19954 ± 0.0107 | 2.77637 ± 0.05598 |
|  | b | 0 | 0 |
|  | R^2^ | 0.99368 | 0.98597 |
|  | Adj. R^2^ | 0.9936 | 0.98557 |
